# Case Report: Interventricular Septal Hematoma Complicating Left Bundle Branch Pacing Lead Implantation

**DOI:** 10.3389/fcvm.2021.744079

**Published:** 2021-09-28

**Authors:** Rujie Zheng, Shengjie Wu, Songjie Wang, Lan Su, Kenneth A. Ellenbogen, Weijian Huang

**Affiliations:** ^1^Department of Cardiology, The First Affiliated Hospital of Wenzhou Medical University, Wenzhou, China; ^2^The Key Lab of Cardiovascular Disease, Science and Technology of Wenzhou, Wenzhou, China; ^3^Virginia Commonwealth University Medical Center, Richmond, VA, United States

**Keywords:** left bundle branch pacing, interventricular septal hematoma, cardiac pacing, His bundle pacing, complication

## Abstract

**Background:** Left bundle branch pacing (LBBP) is a novel physiological pacing and previous studies have confirmed the feasibility and safety of it. The incidence of complications in LBBP is relatively low as reported. Here we present a case of interventricular septal hematoma complicating LBBP lead implantation.

**Case summary:** LBBP was achieved for treatment of high-grade atrioventricular block in a 67-year-old female. Chest pain began 1 h after implantation when the electrocardiogram showed ST-T changes. Then bedside echocardiography confirmed the formation of interventricular septal hematoma. Urgent coronary angiography showed the contrast agent retention and overflow in the interventricular septum. The symptom was relieved half an hour later. Echocardiogram performed 2 h later revealed the size of the hematoma was the same as before. The electrocardiography, coronary angiography and CTA confirmed the resolution of the hematoma at 1-month follow-up. Pacing parameters and cardiac function remained stable during 6-month follow-up.

**Conclusion:** This is the first reported case describing the clinic features and management of interventricular septum hematoma complicating LBBP. The importance of routine echocardiograms after implantation for identifying the hematoma should be highlighted.

## Introduction

His-Purkinje conduction system pacing allows the most physiological left ventricular activation by capturing the conduction bundle and improve clinical outcomes (1, 2). But His bundle pacing (HBP) is limited by a high and unstable threshold, technical difficulties and relatively low success rates (3). Left bundle branch pacing (LBBP) holds promise as a novel modality for physiological pacing that can achieve low and stable pacing parameters and low incidence of complications (4, 5). Here we report a case of the interventricular septal hematoma complicating LBBP lead implantation which has not been reported before.

## Case Report

**Figure 1:**
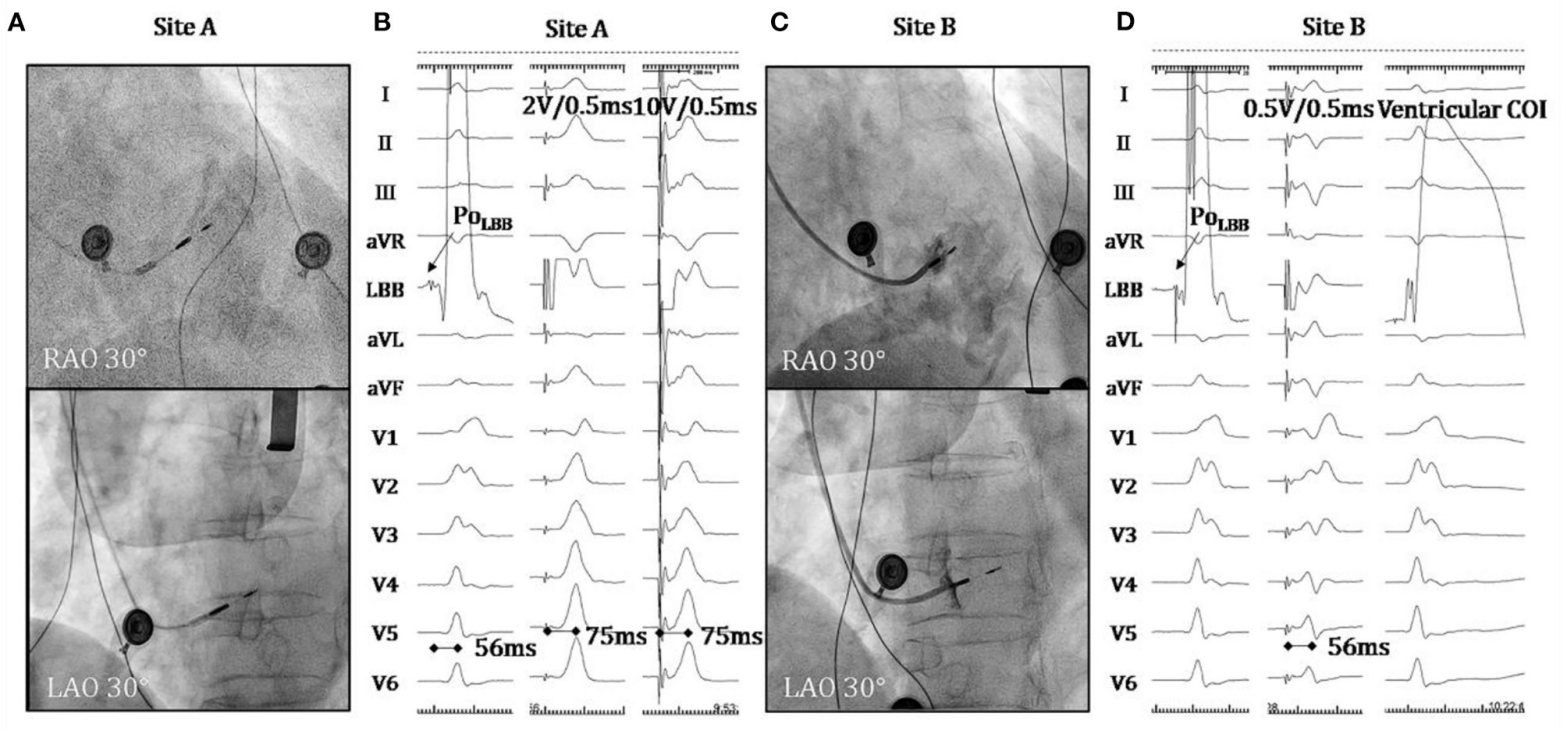
**(A)** Fluoroscopic images at RAO 30° and LAO 30° at the site A which represents where the pacing lead got for the first time. **(B)** The LBB potential noted and the characteristics of paced QRS morphology at the site A. **(C)** Fluoroscopic images at RAO 30° and LAO 30° at the site B. **(D)** The LBB potential noted, the characteristics of paced QRS morphology and ventricular COI at the site B. RAO, right anterior oblique; LAO, left anterior oblique; LBB, left bundle branch; COI, current of injury.

**Figure 2:**
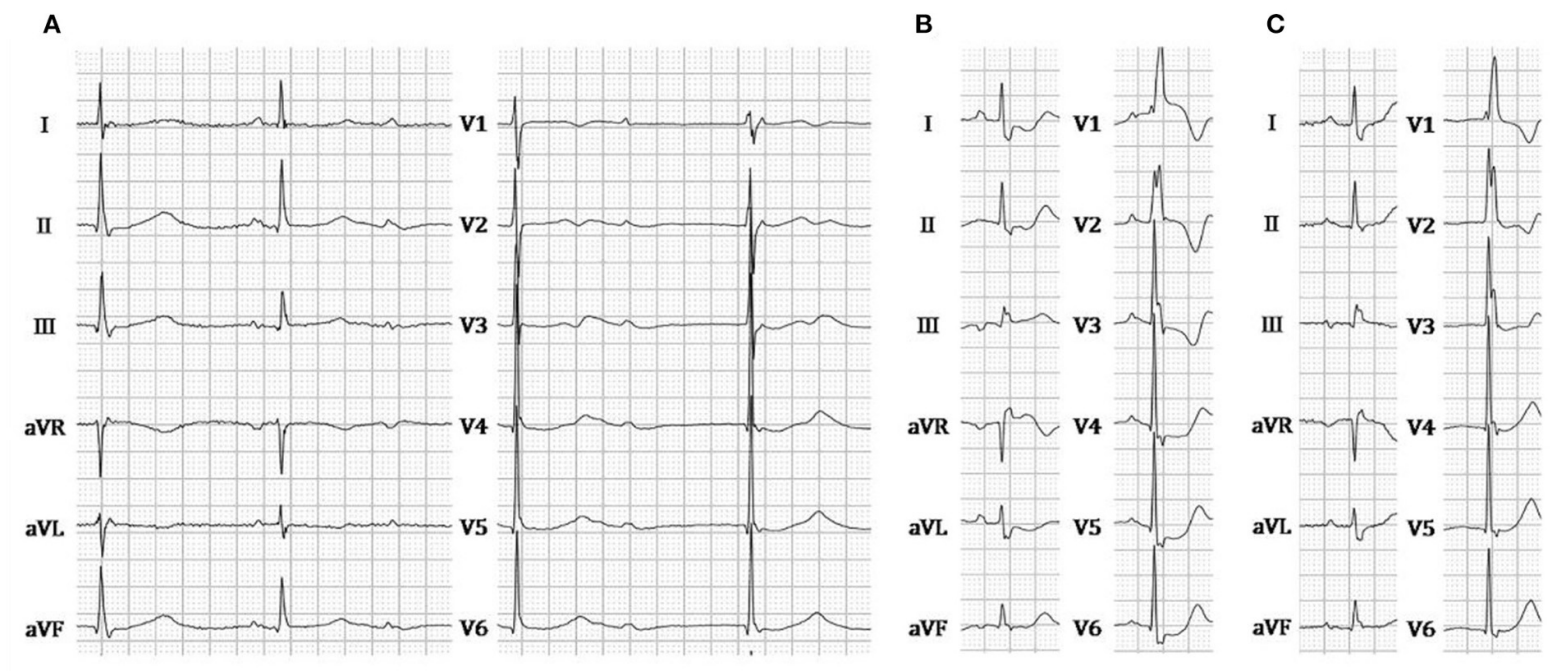
**(A)** ECG during intrinsic rhythm. **(B)** ECG recorded when chest pain occurred. **(C)** ECG recorded when chest pain was relieved. ECG, electrocardiogram.

**Figure 3:**
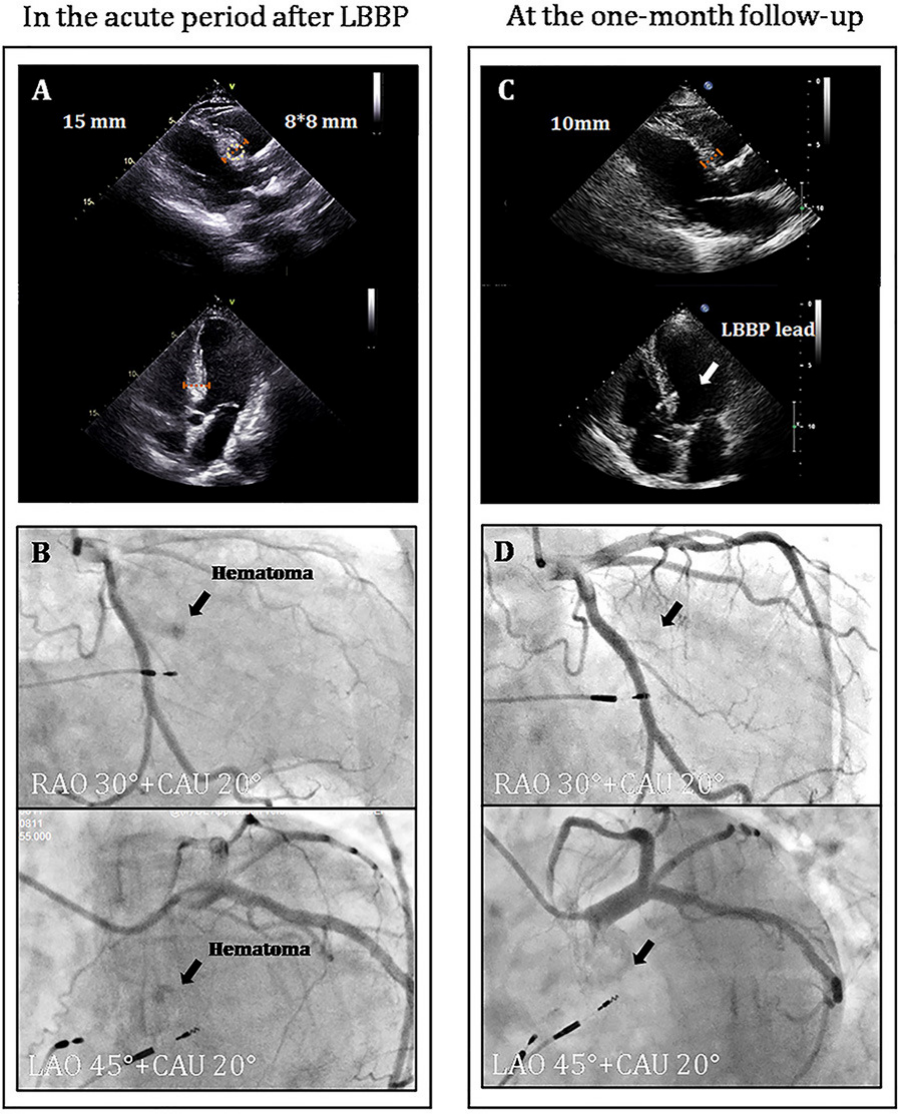
**(A)** The echocardiogram indicated interventricular septal hematoma measuring 8 mm in width. **(B)** Contrast agent retention in the interventricular septum. **(C)** The echocardiography confirmed the hematoma has resolved. **(D)** No contrast agent overflow was found in the interventricular septum. RAO, right anterior oblique; CAU, caudal; LAO, left anterior oblique.

**Figure 4:**
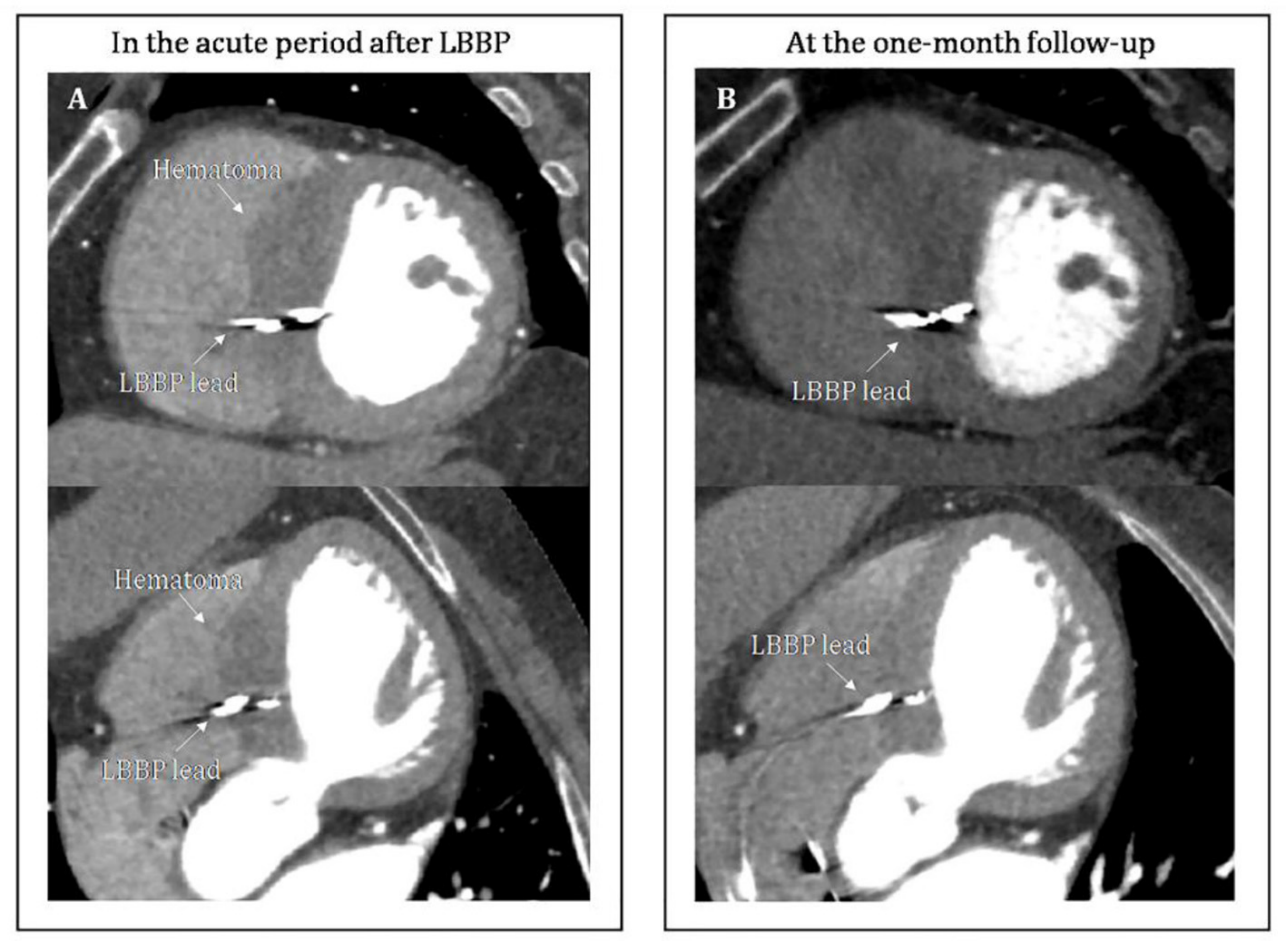
**(A)** CTA showed a hematoma in the basal segment of the interventricular septum in the acute period after LBBP. On non-contrast CTA, a high-density thickening in the interventricular septum was found. Post contrast, no significant change in the enhancement was observed. **(B)** CTA confirmed the hematoma has resolved at the 1-month follow-up. CTA, computed tomography angiography.

A 67-year-old female presenting with dizziness was diagnosed with high-degree atrioventricular block (**Figure 2A**) and referred for pacemaker implantation in August 2020. Her medical history showed hypertension and previous stent implantation for the treatment of left anterior descending artery stenosis with long-term clopidogrel medication. The intracardiac electrogram showed infra-Hisian block, and the His bundle capture threshold was 5 V/0.5 ms. Then, we performed left bundle branch pacing. The lead was advanced into the LBB area located below the septal tricuspid valve. The procedural steps for delivering LBBP and detailed criteria to confirm LBB capture have been described previously, the key point of which is to screw deep enough into the septum (4, 6, 7). At the site A, we failed to capture the LBB (Figures 1A,B) even when we tried to screw deeper where we recorded a smaller LBB potential. Then the lead was moved inferiorly and posteriorly to the site B, LBBP was finally successfully achieved with a larger LBB potential and pacing threshold of 0.5 V/0.5 ms (Figures 1C,D). One hour after the operation, the patient complained of chest pain, and her blood pressure decreased from 131/72 to 96/57 mmHg. The ST-segment depression was recorded during intrinsic rhythm by programming the device to a lower pacing rate (Figure 2B) and pacing parameters remained stable. The bedside echocardiogram revealed an interventricular septal hematoma measuring 8 mm in width without pericardial effusion (Figure 3A). Immediate coronary angiography demonstrated contrast agent overflow and retention in the interventricular septum (IVS) (Figure 3B). The symptoms were relieved quickly, and her blood pressure increased with a normalized ST segment half an hour later (Figure 2C). An echocardiogram was taken again 2 h later, and hematoma progression was not observed. Six hours after implantation, the troponin I level increased from 0.004 to 32.589 μg/L. The next day, computed tomography angiography (CTA) showed a hematoma in the basal segment of the IVS (Figure 4A). After 1 month, echocardiography, CTA confirmed the hematoma had resolved (Figures 3C, 4B). No contrast agent overflow was found in the interventricular septum (Figure 3D) at 1-month follow-up. At 1, 3, and 6 months after implantation, the pacing parameters and LVEF remained stable.

## Discussion

HBP is an ideal strategy to achieve physiological pacing and has been shown to be effective in delivering cardiac resynchronization therapy and improving clinical outcomes (1). With the advancement of technique and devices, the success rate of HBP has significantly improved (8) but is still relatively lower in patients with infranodal block (3). LBBP is a novel pacing strategy with a low and stable threshold firstly reported by Huang et al. (9), which overcomes the limitation of HBP. In infranodal block, LBBP can pace distal to the site of the block with a high success rate and favorable clinical findings (10). Previous studies showed LBBP could improve clinical outcomes in heart failure patients with LBBB (5, 11) and achieve similar improvements in LV function comparable with HBP in patients with CRT indications (5). Recently, a large-scale study has demonstrated LBBP appearing to be a reliable method to achieve satisfying clinical outcomes regardless of pacing indication (12). The low incidence of complications related to LBBP has been confirmed (13), even though complications such as septal perforation and coronary artery injury have been reported (14, 15). Such a large interventricular septal hematoma has not been reported before.

Septal hematoma may occur after coronary intervention treatment, catheter ablation in the interventricular septum, cardiac surgery and chest trauma. The etiology of septal hematoma is unclear and possible explanation is that the injury to the septal perforating arterial branch during the procedure leads to hematoma formation (16). Septal hematoma is a rare complication, but it may result in outflow tract obstruction, conduction abnormalities and cardiac tamponade, which requires immediate cardiac intervention.

Patients with interventricular septal hematoma can present with chest tightness, dyspnea or heart failure symptoms. Electrocardiography can demonstrate ST-T segment depression. This dynamic diffused ST depression can not be fully explained by the local hematoma and ischemia but might also be related to the secondary T wave changes associated with intermittent right bundle branch block, electrotonic modulation of the T wave, hypotension and coronary spasm (17–19). The cardiac troponin levels were significantly greater compared to that following routine LBBP and this increase indicated severe myocardial injury (20). Septal hematoma can be confined to the septum or may cause life-threatening ventricular septal rupture. If the patient has symptoms, ECG abnormalities, or increasing troponin, immediate echocardiography is recommended. A significant widening of the IVS with an internal, hypoechogenic mass may be observed. It is necessary to highlight the importance of routine echocardiography after implantation for identifying small hematomas without any symptoms. Coronary angiography is recommended to further confirm the extent of injury to artery branches and the location of hematoma. CTA is also a useful tool to identify the hematoma. On non-contrast CTA, a high-density thickening in the interventricular septum was found. Post contrast, no significant change in the enhancement was observed. Simultaneously, the relationship of the hematoma and pacing lead could be confirmed by the CTA.

According to the site of LBBP lead and coronary angiography, we thought that the hematoma was caused by the injury to a small branch of the perforating septal artery during the LBBP lead implantation at the site A. The severity of the patient's condition should be comprehensively evaluated and then decisions about the next step of management can be made. In most reports, the patients with septal hematoma usually had uneventful outcomes when treated conservatively particularly if they have stable hemodynamics. Based on the following points: (1) relief of symptoms, stable vital signs and normalized ECG; (2) only small amount of contrast agent overflow and retention; (3) no rupture into the pericardium; (4) good response to conservative treatment in previous reports, we chose conservative treatment and close surveillance. In the next few days, considering that dynamic echocardiogram monitoring demonstrated that the hematoma did not progress, contrast wash-out occurred smoothly and the patient's vital signs remained stable, we thought there was no need for intervention and interruption of mono antiplatelet medication (21). In our case, cardiac function and pacing parameters remained stable at the acute period and during the follow-up. If the hematoma increases in size echoardiographically or even pericardial effusion occurs, antithrombotic therapy should be interrupted, and other approaches would have been considered (22). Surgical treatment with evacuation of the hematoma would have been considered (23). Repeated echocardiography still remains the mainstay of follow-up.

To prevent hematoma formation, a series of evaluations are required. Firstly, anticoagulant use should be carefully assessed. Secondly, always bring awareness to anatomical variants of vessels to avoid injury of potentially important arteries, for example, the sinus of Valsalva aneurysm may compress the septum. In patients with vascular malformation, angiography may be needed to help guide the implantation of pacing lead, but it is not routinely used in clinical practice. Thirdly, the number of the sites for lead implantation should be minimized. To avoid damaging the coronary arteries, especially anterior descending artery and large septal perforator branches, the lead should not be placed too anteriorly at the right anterior oblique fluoroscopic view (4). Fourthly, during the perioperative period, patients' chief complaints should be carefully monitored, such as chest pain, dyspnea or heart failure symptoms. Always pay attention to dynamic changes of pacing parameters and the electrocardiogram during the procedure. Finally, routine echocardiography after the procedure is recommended if there is concern about hematoma formation or expansion. If necessary, coronary angiography could be implemented.

LBBP is generally feasible and safe, but the incidence of the hematoma may be underestimated. Whether the clinical course of the hematoma would be uneventful or not, which requires further personalized management.

## Data Availability Statement

The raw data supporting the conclusions of this article will be made available by the authors, without undue reservation.

## Ethics Statement

Written informed consent was obtained from the individual(s) for the publication of any potentially identifiable images or data included in this article.

## Author Contributions

All authors listed have made a substantial, direct and intellectual contribution to the work, and approved it for publication.

## Funding

This work was supported by the Natural Science for Youth Foundation, grant/award no. 81900345; Key Research and Development Program of Zhejiang (grant no. 2019C03012); Major Project of the Science and Technology of Wenzhou (grant no. ZS2017010).

## Conflict of Interest

The authors declare that the research was conducted in the absence of any commercial or financial relationships that could be construed as a potential conflict of interest.

## Publisher's Note

All claims expressed in this article are solely those of the authors and do not necessarily represent those of their affiliated organizations, or those of the publisher, the editors and the reviewers. Any product that may be evaluated in this article, or claim that may be made by its manufacturer, is not guaranteed or endorsed by the publisher.

## References

[1] Huang W Su L Wu S Xu L Xiao F Zhou X. Long-term outcomes of His bundle pacing in patients with heart failure with left bundle branch block. Heart. (2019) 105:137–43. 10.1136/heartjnl-2018-313415 30093543

[2] Huang W Su L Wu S Xu L Xiao F Zhou X. Benefits of permanent His bundle pacing combined with atrioventricular node ablation in atrial fibrillation patients with heart failure with both preserved and reduced left ventricular ejection fraction. J Am Heart Assoc. (2017) 6:e005309. 10.1161/JAHA.116.005309 28365568 PMC5533020

[3] Vijayaraman P Naperkowski A Ellenbogen KA Dandamudi G. Electrophysiologic insights into site of atrioventricular block: lessons from permanent His bundle pacing. JACC Clin Electrophysiol. (2015) 1:571–81. 10.1016/j.jacep.2015.09.012 29759411

[4] Huang W Chen X Su L Wu S Xia X Vijayaraman P. A beginner's guide to permanent left bundle branch pacing. Heart Rhythm. (2019) 16:1791–6. 10.1016/j.hrthm.2019.06.016 31233818

[5] Wu S Su L Vijayaraman P Zheng R Cai M Xu L. Left bundle branch pacing for cardiac resynchronization therapy: nonrandomized on-treatment comparison with His bundle pacing and biventricular pacing. Can J Cardiol. (2020) 37:319–28 10.1016/j.cjca.2020.04.037 32387225

[6] Vijayaraman P Subzposh FA Naperkowski A Panikkath R John K Mascarenhas V. Prospective evaluation of feasibility, electrophysiologic and echocardiographic characteristics of left bundle branch area pacing. Heart Rhythm. (2019) 16:1774–82 10.1016/j.hrthm.2019.05.011 31136869

[7] Wu S Chen X Wang S Xu L Xiao F Huang Z. Evaluation of the criteria to distinguish left bundle branch pacing from left ventricular septal pacing. JACC Clin Electrophysiol. 7:1166–77. (2021) 10.1016/j.jacep.2021.02.018 33933414

[8] Zanon F Ellenbogen KA Dandamudi G Sharma PS Huang W Lustgarten DL. Permanent His-bundle pacing: a systematic literature review and meta-analysis. Europace. (2018) 20:1819–26. 10.1093/europace/euy058 29701822

[9] Huang W Su L Wu S Xu L Xiao F Zhou X. A novel pacing strategy with low and stable output: pacing the left bundle branch immediately beyond the conduction block. Can J Cardiol. (2017) 33:1736.e1–e3. 10.1016/j.cjca.2017.09.013 29173611

[10] Ye Y Wu S Su L Sheng X Zhang J Wang B. Feasibility and outcomes of upgrading to left bundle branch pacing in patients with pacing-induced cardiomyopathy and infranodal atrioventricular block. Front Cardiovasc Med. (2021) 8:550. 10.3389/fcvm.2021.674452 34195236 PMC8236829

[11] Huang W Wu S Vijayaraman P Su L Chen X Cai B. Cardiac resynchronization therapy in patients with nonischemic cardiomyopathy using left bundle branch pacing. JACC Clin Electrophysiol. (2020) 6:849–58. 10.1016/j.jacep.2020.04.011 32703568

[12] Su L Wang S Wu S Xu L Huang Z Chen X. Long-term safety and feasibility of left bundle branch pacing in a large single-center study. Circ Arrhythmia Electrophysiol. (2021) 14:e009261. 10.1161/CIRCEP.120.009261 33426907

[13] Chen X Wei L Bai J Wang W Qin S Wang J. Procedure-related complications of left bundle branch pacing: a single-center experience. Front Cardiovasc Med. (2021) 8:645947. 10.3389/fcvm.2021.645947 33869306 PMC8044788

[14] Ponnusamy SS Vijayaraman P. Aborted ST-elevation myocardial infarction-An unusual complication of left bundle branch pacing. Heart Rhythm Case Rep. (2020) 6:520–2. 10.1016/j.hrcr.2020.05.010 32817832 PMC7424302

[15] Ravi V Larsen T Ooms S Trohman R Sharma PS. Late-onset interventricular septal perforation from left bundle branch pacing. Heart Rhythm Case Rep. (2020) 6:627–31. 10.1016/j.hrcr.2020.06.008 32983881 PMC7498514

[16] Vargas-Barrón J Roldán FJ Romero-Cárdenas A Molina-Carrión M Vázquez-Antona CA Zabalgoitia M. Dissecting intramyocardial hematoma: clinical presentation, pathophysiology, outcomes and delineation by echocardiography. Echocardiography. (2009) 26:254–61. 10.1111/j.1540-8175.2008.00804.x 19017318

[17] Shan P Huang W Li S Zhou H Dong F Chu M. Acute dysfunction of mechanical aortic valve as electrocardiographic mimic of acute left main coronary artery occlusion. Ann Thorac Surg. (2012) 93:1307–9. 10.1016/j.athoracsur.2011.09.019 22450086

[18] Rosenbaum MB Blanco HH Elizari MV Lázzari JO Davidenko JM. Electrotonic modulation of the T wave and cardiac memory. Am J Cardiol. (1982) 50:213–22. 10.1016/0002-9149(82)90169-2 7102553

[19] Kashou AH May AM DeSimone CV Deshmukh AJ Asirvatham SJ Noseworthy PA. Diffuse ST-segment depression despite prior coronary bypass grafting: an electrocardiographic-angiographic correlation. J Electrocardiol. (2019) 55:28–31. 10.1016/j.jelectrocard.2019.04.014 31078104

[20] Ponnusamy SS Patel NR Naperkowski A Subzposh FA Vijayaraman P. Cardiac troponin release following left bundle branch pacing. J Cardiovasc Electrophysiol. (2021) 32:851–5. 10.1111/jce.14905 33484212

[21] Tompkins C Henrikson CA. Optimal strategies for the management of antiplatelet and anticoagulation medications prior to cardiac device implantation. Cardiol J. (2011) 18:103–9. Available online at: https://journals.viamedica.pl/cardiology_journal/article/view/21290 21305497

[22] Scarparo P Wilschut J Van Mieghem NM Diletti R. Management of septal branch perforation and septal hematoma during retrograde treatment of coronary chronic total occlusion using fat embolization. Can J Cardiol. (2020) 36:966.e15–e17. 10.1016/j.cjca.2019.12.012 32376345

[23] Drago M Butera G Giamberti A Lucente M Frigiola A. Interventricular septal hematoma in ventricular septal defect patch closure. Ann Thorac Surg. (2005) 79:1764–5. 10.1016/j.athoracsur.2003.10.123 15854976

